# Open treatment of ankle fracture as inpatient increases risk of complication

**DOI:** 10.1007/s10195-017-0472-9

**Published:** 2017-10-26

**Authors:** Michelle S. Shen, Ashley C. Dodd, Nikita Lakomkin, Idine Mousavi, Catherine Bulka, A. Alex Jahangir, Manish K. Sethi

**Affiliations:** 1 Vanderbilt Orthopaedic Institute Center for Health Policy, Department of Orthopaedics, Vanderbilt University Medical Center, 1215 21st Avenue South, Suite 4200, Medical Center East, South Tower, Nashville, TN 37232 USA; 2 Department of Biostatistics, Vanderbilt University, Nashville, TN USA

**Keywords:** Ankle fracture, Outpatient, Inpatient, Complications

## Abstract

**Background:**

Ankle fracture is one of the most common injuries treated by orthopaedic surgeons, and its incidence is only expected to rise with an aging population. It is also associated with often costly complications, yet there is little literature on risk factors, especially modifiable ones, driving these complications. The aim of this study is to reveal whether inpatient treatment after ankle fracture is associated with higher incidence of postoperative complications. As the USA moves towards a bundled payment healthcare system, it is imperative that orthopaedists maximize patient outcome and quality of care while also reducing overall costs.

**Materials and methods:**

We used the American College of Surgeons National Surgical Quality Improvement Program database to compare complication rates between inpatient and outpatient treatment of ankle fracture. We collected patient demographics, comorbidities, and postoperative complications from both groups, then compared treatments using a multinomial logistic regression model.

**Results:**

We identified 7383 patients, with 2630 (36%) in the outpatient and 2630 (36%) in the inpatient group. Of these, 104 (4.0%) inpatients compared with 52 (2.0%) outpatients developed a complication (*p* < 0.001).

**Conclusions:**

Inpatients developed major complications including deep wound infection and pulmonary embolism, as well as minor complications such as pneumonia and urinary tract infection, at significantly greater rates. As reimbursement models begin to incorporate value-based care, orthopaedic surgeons need to be aware of factors associated with increased incidence of postoperative complications.

**Level of evidence:**

Level III retrospective comparative study.

## Introduction

Ankle fracture is one of the most common injuries treated by orthopaedic surgeons, occurring at a rate of 187 per 100,000 people [1, 2]. Based on the National Trauma Data Bank, a recent study demonstrated that 55.7% of fractures in the foot and ankle region were ankle fractures [3]. As the population ages, the rate of ankle fracture is rising, leading to more hospital admissions and increased costs [4].

The USA spent US $2.9 trillion, equaling US $9255 per person, on healthcare in 2013, representing a 3.6% increase from 2012 [5]. To contain the rising costs, the USA’s healthcare system is shifting towards a bundled payment model in which complications may not be reimbursed. A study by Avilucea et al. found that there are considerable costs associated with treatment of complications due to isolated ankle fracture [6]. Therefore, complications associated with ankle fracture may become a financial risk for both hospitals and orthopaedic surgeons.

Nevertheless, even with the high rate of ankle fracture, there is minimal literature investigating drivers of complications for these patients. Previous studies have shown specific patient characteristics to be risk factors, for example, American Society of Anesthesiologists (ASA) score, which is a significant predictor of 30-day hospital readmission for orthopaedic trauma injuries [7]. Since ankle fracture patients are commonly treated on both inpatient and outpatient basis depending on the institution, it is essential for surgeons to understand the risk associated with admission status. Orthopaedic studies, including studies of cervical spine fractures, have shown that, in general, outpatient surgery does not increase the rate of postoperative complications [8, 9]. Recent studies, albeit with small sample size and without control for preoperative comorbidities, suggested that outpatient surgery for ankle fracture presents a lower complication rate [10].

Utilizing the American College of Surgeons National Surgical Quality Improvement Program (ACS-NSQIP) database from 2006 to 2013, the aim of this study is to compare the rate of major and minor complications between patients undergoing inpatient and outpatient surgery up to 30 days following surgery.

## Materials and methods

Using a Current Procedural Terminology (CPT) code search, 341,062 orthopaedic patients were identified in the 2006–2013 American College of Surgeons National Surgical Quality Improvement Program (ACS-NSQIP) database. Among these patients, a second CPT code search was used to identify 7383 patients with ankle fracture. Patients were further divided based on hospital admission: 3885 patients underwent inpatient surgery, and 3498 underwent outpatient surgery for ankle fracture. Patient demographics [including age, ASA physical status, body mass index (BMI), sex, and smoking status], preoperative comorbidities [weight loss > 10% in the last 6 months, diabetes, dyspnea, use of steroids, bleeding disorder, on dialysis, functional status, history of chronic obstructive pulmonary disease (COPD), history of congestive heart failure (CHF), and disseminated cancer], and operative characteristics (length of surgery and type of surgical procedure by CPT code) were collected for each patient. Postoperative complications were also evaluated for 30 days following surgery and categorized into minor (superficial wound infection, wound disruption, pneumonia, and urinary tract infection) and major (deep wound infection, organ space infection, myocardial infarction, pulmonary embolism, deep vein thrombosis, sepsis, septic shock, coma, and death) complications.

To investigate whether patients undergoing inpatient or outpatient surgical procedures for ankle fracture presented with similar health profiles, bivariate analyses using the chi-squared test and Wilcoxon–Mann–Whitney test were performed to compare demographics, preoperative comorbidities, operative characteristics, and postoperative complications, as appropriate. Statistical significance was set at *α* = 0.05.

**Fig. 1:**
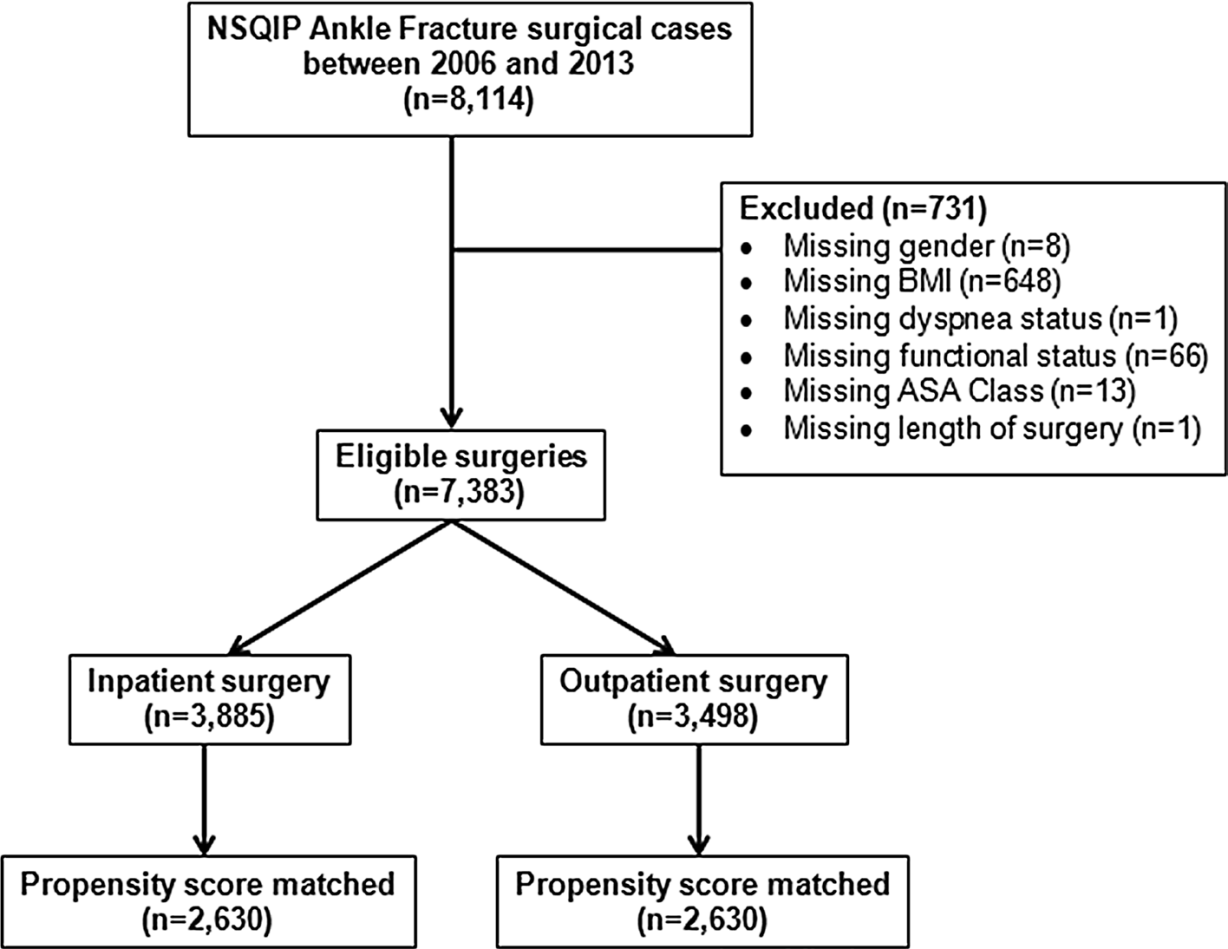
Inclusion and exclusion criteria

To control for confounding variables, we utilized a propensity score matched model, the advantages of which have been previously demonstrated [11–13]. In general, propensity score matching adjusts for differences in individual patient characteristics, such as demographics and comorbidities, to more accurately assess the outcome due to treatment. We calculated the propensity of having outpatient surgery based on patient demographics, preoperative comorbidities, and surgical intervention. Therefore, patients who were ineligible to receive outpatient surgery due to comorbidities or injury severity were removed from the analysis. We then matched patients undergoing inpatient surgery to patients undergoing outpatient surgery based on their propensity scores (Fig. 1) using an 8-to-1 greedy matching algorithm in 1:1 ratio. After matching, there was no significant difference between the CPT codes of the inpatient versus outpatient population, suggesting similar types of ankle fracture between groups (Table 2). Rates of minor, major, and total complications were evaluated using bivariate analysis after propensity scoring the cohort. A multinomial logistic regression model was used to assess the odds of minor and major postoperative complications within 30 days after surgery, adjusting for surgical duration.

Obtaining informed consent from involved patients was waived by an Institutional Review Board. All procedures involving human participants were in accordance with the 1964 Helsinki Declaration and its later amendments. This study was approved by an Institutional Review Board.

## Results

Prior to matching patients based on propensity score, inpatients were shown to be significantly older and to have higher BMI compared with outpatients (*p* < 0.001). Inpatients also presented with a significantly higher rate of preoperative comorbidities, including dyspnea, steroid use, total functional dependency, history of bleeding disorder, dialysis use, history of COPD, history of CHF, and disseminated cancer (*p* < 0.001). Additionally, as shown in Table 1, 16.6% (*n* = 645) of inpatients had diabetes compared with only 7.2% (*n* = 250) of outpatients (*p* < 0.001).

**Table 1:** Patient characteristics among surgically treated ankle fracture patients by admission status before propensity score matching

	Inpatient	Outpatient	*p* value
	(*N* = 3885)	(*N* = 3498)	
Patient demographics			
Age (years), median (IQR)	56 (41–69)	45 (31–57)	<0.001
ASA class, median (IQR)	2 (2–3)	2 (1–2)	<0.001
Body mass index (kg/m^2^), median (IQR)	29.8 (26.0–34.8)	28.8 (25.4–33.4)	<0.001
Male, *N* (%)	1383 (35.6)	1700 (48.6)	<0.001
Smoker, *N* (%)	874 (22.5)	1033 (29.5)	<0.001
Preoperative characteristics			
Weight loss > 10% in last 6 months, *N* (%)	6 (0.2)	2 (0.1)	0.205
Diabetic, *N* (%)	645 (16.6)	250 (7.2)	<0.001
Dyspnea, *N* (%)			<0.001
No	3687 (94.9)	3408 (97.4)	
With moderate exertion	179 (4.6)	86 (2.5)	
At rest	19 (0.5)	4 (0.1)	
Use of steroids, *N* (%)	100 (2.6)	40 (1.1)	<0.001
Bleeding disorder, *N* (%)	222 (5.7)	60 (1.7)	<0.001
On dialysis, *N* (%)	39 (1.0)	3 (0.1)	<0.001
Functional status, *N* (%)			<0.001
Independent	3490 (89.8)	3403 (97.3)	
Partially dependent	378 (9.7)	93 (2.7)	
Totally dependent	17 (0.4)	2 (0.1)	
History of COPD, *N* (%)	189 (4.9)	51 (1.5)	<0.001
History of CHF, *N* (%)	35 (0.9)	6 (0.2)	<0.001
Disseminated cancer, *N* (%)	13 (0.3)	2 (0.1)	0.008
Operative characteristics			
CPT code, *N* (%)			<0.001
27766	188 (4.8)	282 (8.2)	
27784	74 (1.9)	46 (1.3)	
27792	878 (22.6)	1298 (37.1)	
27814	1494 (38.5)	1014 (29.0)	
27822	838 (21.6)	517 (14.8)	
27823	260 (6.7)	109 (3.1)	
27829	153 (3.9)	229 (6.6)	
Length of surgery (min), median (IQR)	70 (50–99)	62 (44–85)	<0.001
Propensity score, median (IQR)	0.43 (0.27–0.55)	0.56 (0.46–0.67)	<0.001

**Fig. 2:**
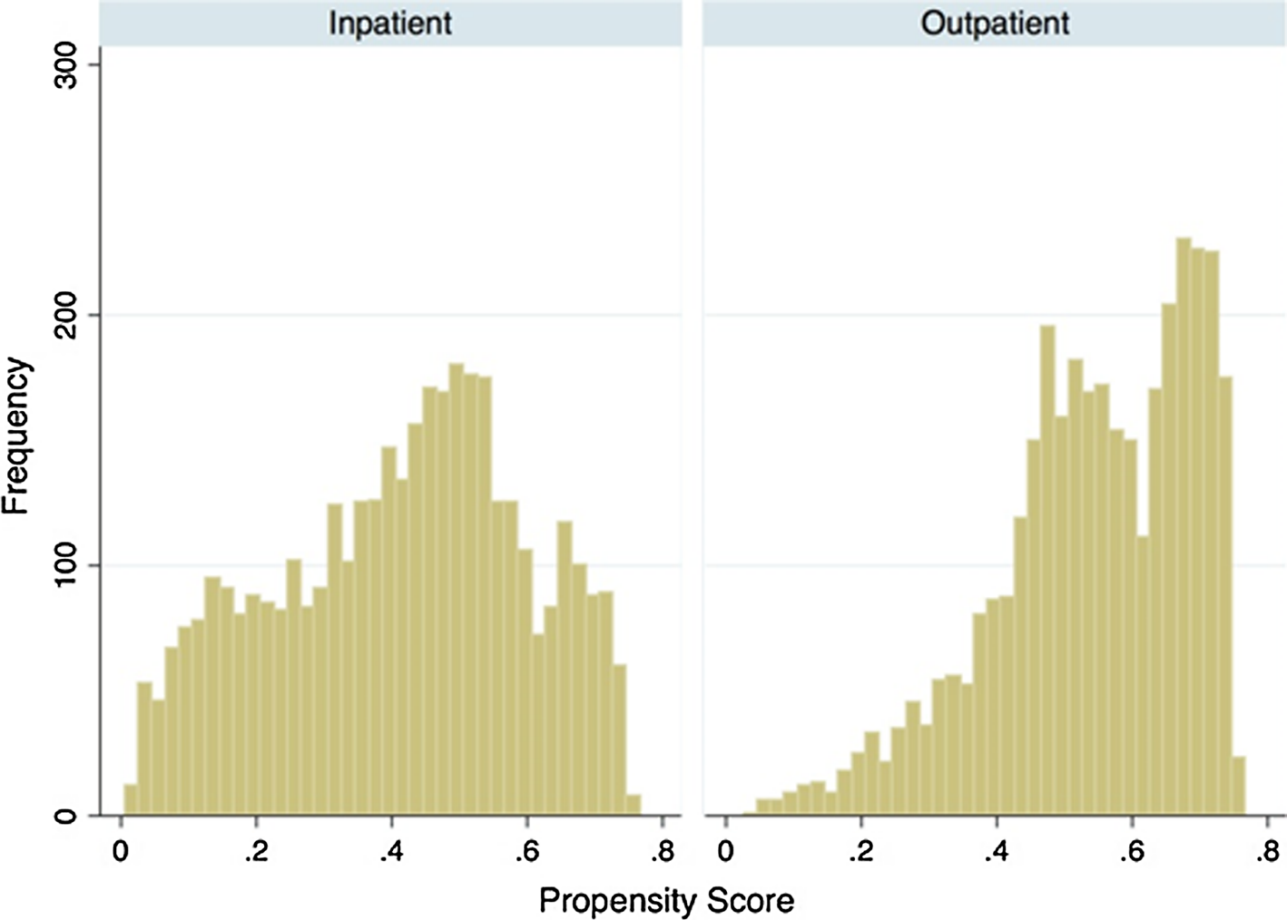
Histograms of propensity score by admission status before matching

**Fig. 3:**
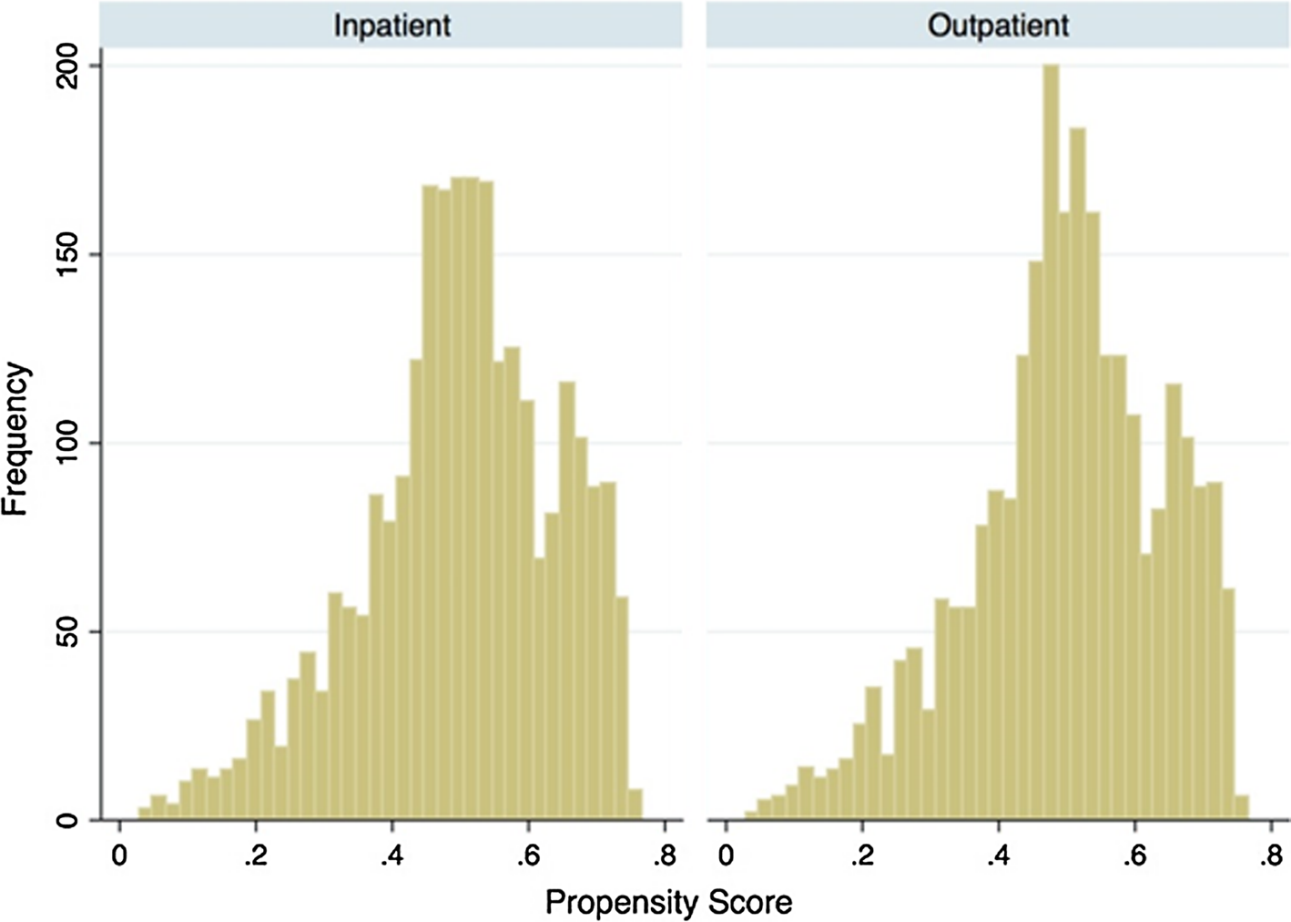
Histograms of propensity score by admission status after matching

**Fig. 4:**
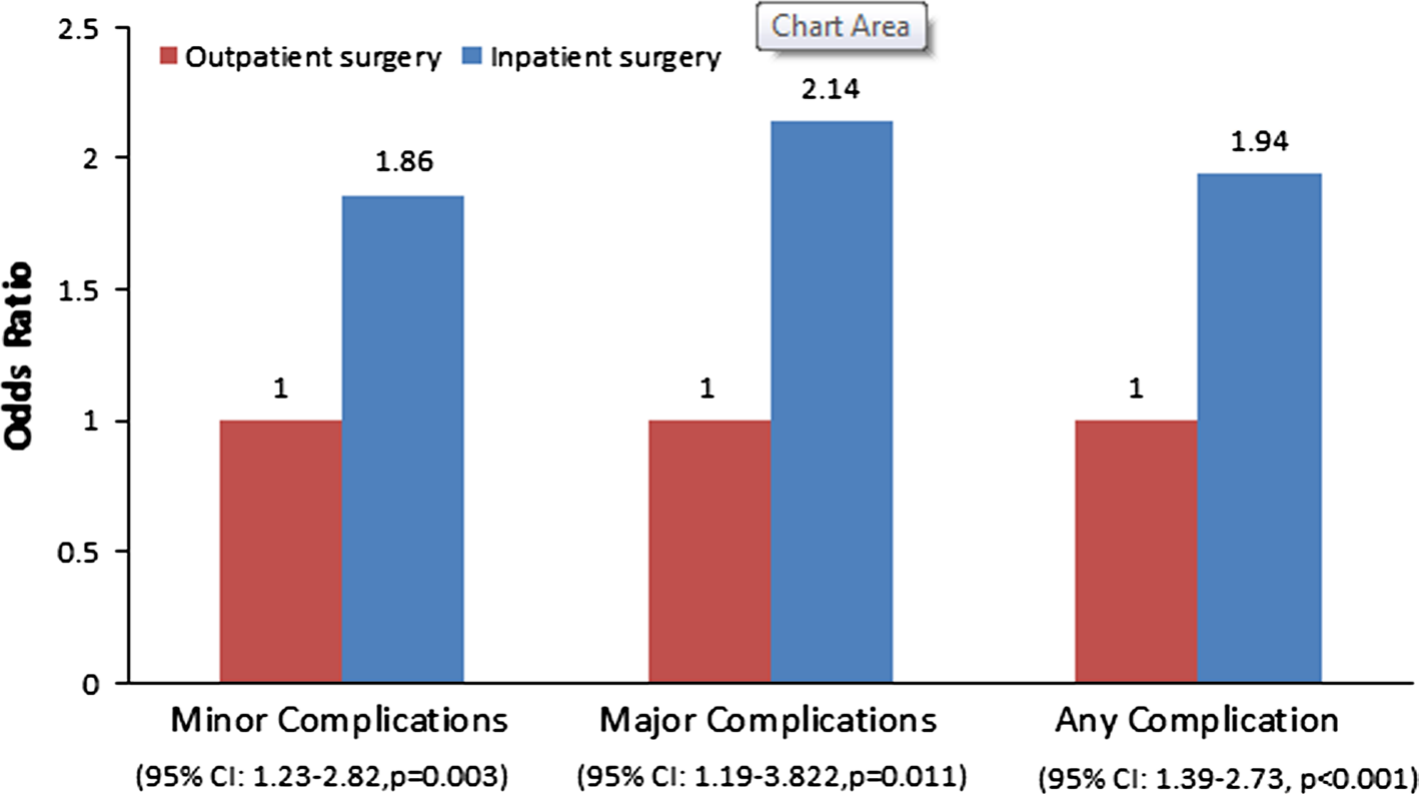
Multivariate analysis of complications by admission status

To control for the increased rate of preoperative comorbidities faced by inpatients, the inpatient and outpatient cohorts were propensity score matched, as shown in Fig. 1, according to which 2630 (36%) outpatients were matched to 2630 (36%) inpatients. Figures 2 and 3 present histograms of inpatients and outpatients before and after propensity matching, respectively. The distribution of propensity scores between outpatient and inpatient cases was more similar after matching, suggesting that these cases are similar in terms of individual patient characteristics and demographics. There were no significant differences in patient demographics, such as age, BMI, ASA class, gender or smoking habits, between the inpatient and outpatient cohorts following propensity score matching. Additionally, since all patients were matched based on preoperative comorbidities, inpatients and outpatients did not significantly vary in their level of health prior to surgery (Table 2). However, length of surgery was significantly longer for inpatients (median: 69 min, IQR: 49–98 min) compared with outpatients (median: 65 min, IQR: 45–88 min) (*p* < 0.001) (Fig. 4).

**Table 2:** Patient characteristics among surgically treated ankle fracture patients by admission status after propensity score matching

	Inpatient	Outpatient	*p* value
	(*N* = 2630)	(*N* = 2630)	
Patient demographics			
Age (years), median (IQR)	63 (49–74)	63 (48–74)	0.707
ASA class, median (IQR)	2 (2–2)	2 (2–2)	0.430
Body mass index (kg/m^2^), median (IQR)	29.3 (25.8–34.0)	29.3 (25.8–34.2)	0.957
Male, *N* (%)	1108 (42.1)	1090 (41.4)	0.615
Smoker, *N* (%)	706 (26.8)	725 (27.6)	0.556
Preoperative characteristics			
Weight loss >10% in last 6 months, *N* (%)	0 (0.0)	2 (0.1)	0.157
Diabetic, *N* (%)	238 (9.1)	241 (9.2)	0.886
Dyspnea, *N* (%)			0.652
No	2561 (97.4)	2554 (97.1)	
With moderate exertion	67 (2.6)	72 (2.7)	
At rest	2 (0.1)	4 (0.2)	
Use of steroids, *N* (%)	41 (1.6)	37 (1.4)	0.648
Bleeding disorder, *N* (%)	60 (2.3)	59 (2.2)	0.926
On dialysis, *N* (%)	6 (0.2)	3 (0.1)	0.317
Functional status, *N* (%)			0.458
Independent	2520 (95.8)	2535 (96.4)	
Partially dependent	106 (4.0)	93 (3.5)	
Totally dependent	4 (0.2)	2 (0.1)	
History of COPD, *N* (%)	56 (2.1)	51 (1.9)	0.625
History of CHF, *N* (%)	7 (0.3)	6 (0.2)	0.781
Disseminated cancer, *N* (%)	0 (0.0)	2 (0.1)	0.157
Operative characteristics			
CPT code, *N* (%)			0.919
27766	163 (6.2)	157 (6.0)	
27784	44 (1.7)	45 (1.7)	
27792	749 (28.5)	722 (27.5)	
27814	915 (34.8)	958 (36.4)	
27822	520 (19.8)	505 (19.2)	
27823	103 (3.9)	109 (4.1)	
27829	136 (4.9)	134 (5.1)	
Length of surgery (min), median (IQR)	69 (49–98)	65 (45–88)	<0.001
Propensity score, median (IQR)	0.51 (0.42–0.60)	0.51 (0.42–0.60)	0.9283

After matching patients based on preoperative health conditions and other confounding variables, there was a significant difference in the complication rates for the inpatient and outpatient groups with ankle fracture (Table 3). Overall, 104 (4.0%) inpatients compared with 52 (2.0%) outpatients developed any complication (*p* < 0.001), while 1.7% (*n* = 45) of inpatients compared with 0.8% (*n* = 20) of outpatients developed a major complication (*p* = 0.002), including higher rates of deep wound infection (*p* = 0.032) and pulmonary embolism (*p* = 0.004). Inpatients also presented a significantly higher rate of minor complications (*p* = 0.001), including superficial wound infection (*p* = 0.026), pneumonia (*p* = 0.014), and urinary tract infection (*p* = 0.027).

**Table 3:** Complication rates among surgically treated ankle fracture patients by admission status after propensity score matching

	Inpatient	Outpatient	*p* value
	(*N* = 2630)	(*N* = 2630)	
Minor complications, *N* (%)			
Superficial wound infection	34 (1.3)	18 (0.7)	0.026
Wound disruption	8 (0.3)	11 (0.4)	0.491
Pneumonia	6 (0.2)	0 (0.0)	0.014
Urinary tract infection	24 (0.9)	11 (0.4)	0.027
Any minor complication	68 (2.6)	35 (1.3)	0.001
Major complications, *N* (%)			
Deep wound infection	11 (0.4)	3 (0.11)	0.032
Organ space infection	4 (0.2)	2 (0.1)	0.414
Myocardial infarction	1 (0.0)	1 (0.0)	1.000
Pulmonary embolism	13 (0.5)	2 (0.1)	0.004
Deep vein thrombosis	13 (0.5)	7 (0.3)	0.179
Sepsis	7 (0.3)	3 (0.1)	0.205
Septic shock	1 (0.0)	0 (0.0)	0.317
Coma	0 (0.0)	1 (0.0)	0.317
Death within 30 days	2 (0.1)	3 (0.1)	0.655
Any major complication	45 (1.7)	20 (0.8)	0.002
Overall complications, *N* (%)	104 (4.0)	52 (2.0)	<0.001

Inpatients were 1.94 times more likely to develop a complication compared with outpatients. Specifically, inpatients were 2.14 times more likely to develop a major complication and 1.86 times more likely to develop a minor complication compared with their outpatient counterparts (Table 4).

**Table 4:** Chi-squared analysis for CPT code distribution between inpatient and outpatient cohorts

	Value	*df*	Asymptotic significance (two-sided)
Pearson chi squared	2.011	6	0.919
Likelihood ratio	2.011	6	0.919
No. of valid cases	5260		

## Discussion

This study is the first to report that inpatient surgery for ankle fracture may lead to increased risk of minor, major, and all complications 30 days following surgery even after propensity score matching. Patients undergoing inpatient surgery were approximately twice as likely to develop any complication as those undergoing outpatient surgery (*p* < 0.001). Previously, other studies have used the propensity score model to compare the rate of postoperative complications between inpatients and outpatients. Qin et al. [14] utilized the ACS-NSQIP database to evaluate the risk of postoperative complications in outpatient versus inpatient surgery for tissue expander (TE)-based reconstruction following mastectomy by first stratifying patients via admission status and then propensity score matching. Khavanin et al. [15] also applied propensity score matching to show that, compared with outpatients, inpatients undergoing thyroidectomy presented with higher rates of postoperative complications.

Surgical intervention for ankle fracture generally has positive outcomes, yet certain risk factors for complications and poor outcomes have been investigated [16–20]. Since approximately 25% of ankle fractures are treated with surgical stabilization in the USA, it is essential for orthopaedic surgeons to understand the drivers of postoperative complications [21, 22]. A study by Weckback et al. which used a prospective database to analyze the rate of postoperative complications and need for surgical revision depending on outpatient or inpatient care for isolated ankle fracture similarly demonstrated that inpatient surgery presented a significantly higher complication rate [10]. That study also found the rate of complications to be 3.1% for outpatients and 9.1% for inpatients, which is higher than the data in our study. Unlike Weckback’s study, however, our investigation included a larger cohort of ankle fracture patients (7383 patients compared with 476 patients, respectively) and controlled for preoperative comorbidities and patient demographics by propensity score matching of patients.

We found that superficial wound infections were significantly more common for inpatients at a rate of 1.3% compared with 0.7% for outpatients (*p* = 0.026). Wound complications, such as superficial wound infections, are found to represent a major risk for all patients undergoing ankle fracture surgery. It is essential to note that our study controlled for preoperative comorbidities, such as diabetes and smoking, by propensity score matching of patients. Diabetes has been shown to drastically increase the risk of postoperative infections after surgical treatment of ankle fracture, while smoking has been found to impede wound healing [20, 23]. Because we controlled for these comorbidities, based on our results, it can be concluded that a driver of minor wound complications, such as superficial infections, may be associated with increased time spent in hospital as experienced by inpatients.

Patients undergoing inpatient surgery for ankle fracture also presented with a significantly higher rate of pneumonia (*p* = 0.014) and urinary tract infection (*p* = 0.027) compared with outpatients within 30 days following surgery. Postoperative pneumonia and urinary tract infection are considered two out of the three most common infectious complications for surgical inpatients [24]. Our study therefore allows surgeons and hospitals to evaluate the effect that inpatient surgery has on the development of such common postoperative complications.

When investigating major postoperative complications within 30 days, inpatient surgery was associated with increased risk of high rate of deep wound infection (*p* = 0.032) and pulmonary embolism (*p* = 0.004) compared with outpatient surgery. The study by Miller et al. found that, of 478 ankle fracture patients, 1.25% returned to the operating room for wound-related complications. These results highlight the importance of discussing potential wound-related complications with patients before admitting them to an inpatient service [22]. In particular, orthopaedic surgeons must consider a patient’s preoperative comorbidities, such as diabetes, before admitting them for inpatient ankle fracture surgery due to the risk of increasing complications [22]. Additionally, increased risk of pulmonary embolism has been shown in other studies investigating risks associated with inpatient surgery [14]; For example, Khavanin et al. found that inpatients undergoing thyroidectomy had twice the risk of developing pulmonary embolism compared with outpatients [15]. Surgical intervention for ankle fracture, in general, has also been shown to increase the risk of pulmonary embolism; therefore, orthopaedic surgeons should assess the patient for their risk of developing such complication before treating them as an inpatient [25].

Our results must be interpreted in the context of the limitations of this study. First, since we used the NSQIP database, we were limited by the number of patients and the risk factors evaluated. Additionally, NSQIP data do not specify isolated ankle fractures, therefore further prospective research is needed to evaluate complication rates for isolated ankle fracture. The NSQIP database also does not specify certain features of each patient’s treatment. We do not know how long after injury the surgical procedures were performed, whether patients were given oral or injected thrombosis prophylaxis, whether patients received antibiotic prophylaxis, which type of implant the patient received, or whether tourniquets were used on the patients. The NSQIP database was also limiting due to the designation of inpatient and outpatient status. Inpatients were defined as patients who stayed in the hospital longer than 23 h without differentiating between same-day and overnight stay. Medicare, for example, has an “observational status” in which patients can be admitted for up to 2 days as outpatients, therefore such policies could lead to mislabeling of outpatients and inpatients within the NSQIP database [26]. Due to the anonymity of the database, the study design could not account for variability between hospital policies.

Our study found that outpatient surgery for ankle fracture is associated with decreased risk for developing 30-day postoperative complications. Even when controlling for comorbid conditions, inpatients presented with higher rates of minor and major complications. Multivariate analysis corroborated inpatient status as an independent risk factor for such a complication. Orthopaedic surgeons should perhaps consider treating ankle fractures using outpatient procedures if given the option. In a future bundled payment system, orthopaedic trauma surgeons need to be aware of the factors influencing complications to decrease cost and improve patient care.

## References

[1] Centers for Medicare and Medicaid Services. Baltimore (MD). National Health Expenditures 2013 Highlights (2013) http://www.cms.gov/Research-Statistics-Data-and-Systems/Statistics-Trends-and-Reports/NationalHealthExpendData/downloads/highlights.pdf. Accessed 9 Apr 2015

[2] Daly PJ, Fitzgerald RH, Melton LJ, Ilstrup DM (1987). Epidemiology of ankle fractures in Rochester, Minnesota. Acta Orthop Scand.

[3] Salai M, Dudkiewicz I, Novikov I, Amit Y, Chechick A (2000). The epidemic of ankle fractures in the elderly—is surgical treatment warranted?. Arch Orthop Trauma Surg.

[4] Shibuya N, Davis ML, Jupiter DC (2014). Epidemiology of foot and ankle fractures in the United States: an analysis of the National Trauma Data Bank (2007 to 2011). J Foot Ankle Surg.

[5] Thur CK, Edgren G, Jansson KA, Wretenberg P (2012). Epidemiology of adult ankle fractures in Sweden between 1987 and 2004: a population-based study of 91,410 Swedish inpatients. Acta Orthop.

[6] Avilucea FR, Greenberg SE, Grantham WJ (2014). The costs of operative complications for ankle fractures: a case control study. Adv Orthop.

[7] Sathiyakumar V, Molina CS, Thakore RV, Obremskey WT, Sethi MK (2015). ASA score as a predictor of 30-day perioperative readmission in patients with orthopaedic trauma injuries: an NSQIP analysis. J Orthop Trauma.

[8] Lee MJ, Kalfas I, Holmer H, Skelly A (2014). Outpatient surgery in the cervical spine: is it safe?. Evid Based Spine Care J.

[9] Andrés-Cano P, Godino M, Vides M, Guerado E (2015). Postoperative complications of anterior cruciate ligament reconstruction after ambulatory surgery. Rev Esp Cir Octop Traumatol.

[10] Weckbach S, Flierl MA, Huber-Lang M, Gebhard F, Stahel PF (2011). Surgical treatment of ankle fractures as an outpatient procedure. A safe and resource-efficient concept?. Unfallchirurg.

[11] Austin PC (2009). Some methods of propensity-score matching had superior performance to others: results of an empirical investigation Monte Carlo simulations. Biom J.

[12] Austin PC (2011). Optimal caliper widths for propensity-score matching when estimating differences in means and differences in proportions in observational studies. Pharm Stat.

[13] Austin PC (2014). A comparison of 12 algorithms for matching on the propensity score. Stat Med.

[14] Qin C, Antony AK, Aggarwal A, Jordan S, Gutowski KA, Kim JY (2015). Assessing outcomes and safety of inpatient versus outpatient tissue expander immediate breast reconstruction. Ann Surg Oncol.

[15] Khavanin N, Mlodinow A, Kim JY (2015). Assessing safety and outcomes in outpatient versus inpatient thyroidectomy using the NSQIP: a propensity score matched analysis of 16,370 patients. Ann Surg Oncol.

[16] Bibbo C, Lin SS, Beam HA, Behrens FF (2001). Complications of ankle fractures in diabetic patients. Orthop Clin N Am.

[17] Flynn JM, Rodriguez-del Rio F, Pizá PA (2000). Closed ankle fractures in the diabetic patient. Foot Ankle Int.

[18] Pagliaro AJ, Michelson JD, Mizel MS (2001). Results of operative fixation of unstable ankle fractures in geriatric patients. Foot Ankle Int.

[19] Scott AM (2010). Diagnosis and treatment of ankle fractures. Radiol Technol.

[20] SooHoo NF, Krenek L, Eagan MJ, Gurbani B, Ko CY, Zingmond DS (2009). Complication rates following open reduction and internal fixation of ankle fractures. J Bone Joint Surg Am.

[21] Ganesh SP, Pietrobon R, Cecílio WA, Pan D, Lightdale N, Nunley JA (2005). The impact of diabetes on patient outcomes after ankle fracture. J Bone Joint Surg Am.

[22] Miller AG, Margules A, Raikin SM (2012). Risk factors for wound complications after ankle fracture surgery. J Bone Joint Surg Am.

[23] Wukich DK, Lowery NJ, McMillen RL, Frykberg RG (2010). Postoperative infection rates in foot and ankle surgery: a comparison of patients with and without diabetes mellitus. J Bone Joint Surg Am.

[24] Wren SM, Martin M, Yoon JK, Bech F (2010). Postoperative pneumonia-prevention program for the inpatient surgical ward. J Am Coll Surg.

[25] Kadous A, Abdelgawad AA, Kanlic E (2012). Deep venous thrombosis and pulmonary embolism after surgical treatment of ankle fractures: a case report and review of literature. J Foot Ankle Surg.

[26] Medicare.gov. Are you a hospital inpatient or outpatient? (2014) https://www.medicare.gov/Pubs/pdf/11435.pdf. Accessed 9 Apr 2015

